# Prevalence of Subclinical Hypothyroidism in Acute Coronary Syndrome in Nondiabetics: Detailed Analysis from Consecutive 1100 Patients from Eastern India

**DOI:** 10.1155/2018/9030185

**Published:** 2018-09-04

**Authors:** Sudeb Mukherjee, Suhana Datta, S. C. Mandal

**Affiliations:** ^1^Department Of Cardiology, ICVS, IPGME&R, Kolkata, West Bengal, India; ^2^R.I.O., Medical College, Kolkata, West Bengal, India

## Abstract

**Background:**

The association between subclinical thyroid dysfunction (defined by no symptoms or clinical features of hypothyroidism but biochemically TSH level in the range of above 5 miu/ml but below 10 miu/ml with normal FT4 level) and Acute Coronary Syndrome (ACS) is not known so far. This study was done to calculate the prevalence of subclinical thyroid dysfunction in patients with ACS.

**Methods:**

A retrospective chart review of 1100 consecutive patients was done who presented to Emergency Department with symptoms suggestive of ACS and admitted. They were later classified in 3 categories that includes Acute ST Elevated Myocardial Infarction (STEMI), Unstable Angina (UA), and Acute Non-ST Elevated Myocardial Infarction (NSTEMI). Thyroid function test (FT4, TSH) and antithyroid peroxidase (TPO) were done and evaluated properly.

**Results:**

Of 1100 consecutive patients 168 (15.27%) patients had the biochemical features of subclinical thyroid dysfunction. These 168 patients include 60 STEMI, 66 NSTEMI, and 42 Unstable Angina patients. There were no statistically significant differences in terms of left ventricular ejection fraction (LVEF) and catheterisation results considering thyroid dysfunction.

**Conclusions:**

Subclinical thyroid dysfunction is quite prevalent in ACS patients. There are no significant associations between STEMI, Unstable Angina, or NSTEMI patients in terms of thyroid dysfunction neither in single vessel versus multivessel disease involvement. The causative role and outcomes of treatment are still uncertain and need further follow-up.

## 1. Introduction

The association between thyroid dysfunction and acute coronary syndrome still remains elusive. Thyroid hormones have permissive role in almost all metabolic actions in the body. Therefore alterations in the functions of hormone may lead to several derangements. Thyroid hormone function has great impact on cardiovascular physiology that includes heart rate, blood pressure, cardiac output, systemic vascular resistance, and myocardial contractility [1]. Subclinical hypothyroidism has been associated with increased incidence of atherosclerosis and myocardial infarction in several studies [2]. Presence of antithyroid peroxidase (TPO) antibody indicates heightened risk [3]. Recent study from UK has also reported that treating patient of subclinical hypothyroidism with thyroxine replacement may decrease incidence of ischaemic heart disease events and cardiovascular mortality [4]. Subclinical hypothyroidism increases isovolumetric relaxation time, decreases endothelial relaxation, and decreases cardiac contractility [5]. These effects are very important in the settings of acute coronary syndrome where function of parts of myocardium is impaired due to ischaemia related injury. Mild to moderate pericardial effusion which may also occur during ACS is also seen in cases of subclinical hypothyroidism [6]. Whether replacement with levothyroxine in these acute settings will improve the overall outcome or not is debatable issue.

Till now there is no data of thyroid dysfunction prevalence in ACS patients. This study was done to look for the overall prevalence in large scale basis and whether there is any association in terms of severity of disease process.

## 2. Aims and Objectives

(1) To assess the thyroid abnormalities in ACS patients.

(2)To assess the anti-TPO positivity in patients with thyroid dysfunction.

(3) To assess any association between STEMI, NSTEMI, and Unstable Angina in terms of LVEF, angiographic appearance, and overall outcomes.

## 3. Materials and Methods

The study includes 1100 consecutive patients of ACS who attended Emergency Department with typical symptoms. ECG were done; echocardiographic assessment and biochemical parameter of myocardial injury (Troponin T) were done. The patients were admitted with diagnosis of STEMI, NSTEMI, and Unstable Angina according to reports. Thyroid function test (FT4, TSH) was done on admission anti-TPO antibody tests were conducted with the same blood sample. All patients underwent coronary angiogram (CAG) followed by revascularization where applicable. All data were collected and analysed accordingly.

## 4. Exclusion Criteria

1. Patients of diabetes mellitus.
2. Age more than 75 years.
3. Proven thyroid disorder and under treatment.
4. Very sick or critically injured patients.
5. Patients who had undergone surgery of the thyroid gland.

## 5. Data Analysis

Data analysis has been done in SPSS software, Chi square test was used in case of nonparametric value, and p value of <0.05 is considered significant.

## 6. Results and Analysis

Total number of ACS patients were 1100. Of total 1100 patients 706 were male (64.18%) and 394 were female (35.81%). Baseline demographics have been shown in Table 1. Out of 1100 patients 493 patients (44.81%) were of ST Elevated Myocardial Infarction (STEMI), 403 patients (36.63%) were of Non-ST Elevated Myocardial Infarction (NSTEMI) and 204 patients (18.54%) were of Unstable Angina (UA). Mean age of the patient population was 62.34 years (SD- 2.6), 58.98 years (SD-3.1), and 53.76 years (SD-1.6) for STEMI, NSTEMI, and UA group, respectively. Male population was more in number in each group except in UA where female population was numerically little bit more. Almost all patients presented to Emergency Department with complaints of chest pain. Hypertension was found to be associated more with UA although statistically nonsignificant with P value of 0.56. Average blood pressure in each group was 140/92 mm of Hg, 130/90 mm of Hg, and 150/85 mm of Hg in STEMI, NSTEMI, and UA group, respectively. Almost all patients presented with heart rate more than 100/minutes. Neither bradycardia nor diastolic hypertension was noted in this study. 63.5% patients in the STEMI group admitted with haemodynamic instability status compared to 23% in UA group and NSTEMI in 41% patients. Left Ventricular Ejection Fraction (LVEF) between different groups before intervention showed the mean value of 46.54% (SD-5.81, SE-1.01) in the STEMI group, 49.43% (SD-5.44, SE-1.01) in the NSTEMI group, and 51.83% (SD- 6.21, SE-1.11) in the UA group. Of the all study population, 50-80% had anterior wall involvement with preponderance of multivessel involvement. Distribution of patients according to coronary angiogram findings (%) has been shown in Table 2. LDL cholesterol and triglyceride were found to be high in all groups. Average LDL cholesterol value was found to be more than 120 mg/dl in all groups. A large number of patients were smoker in this study.

The study revealed that 45.72% of patients (503 out of 1100) have the biochemical features of thyroid dysfunction. Out of total 503 patients 334 patients have the biochemical features of hypothyroidism (66.40% of total thyroid dysfunction and 30.36% of total population) and 169 patients have biochemical features suggestive of hyperthyroidism (33.59% of total thyroid dysfunction and 15.36% of total population).

**Figure 1:**
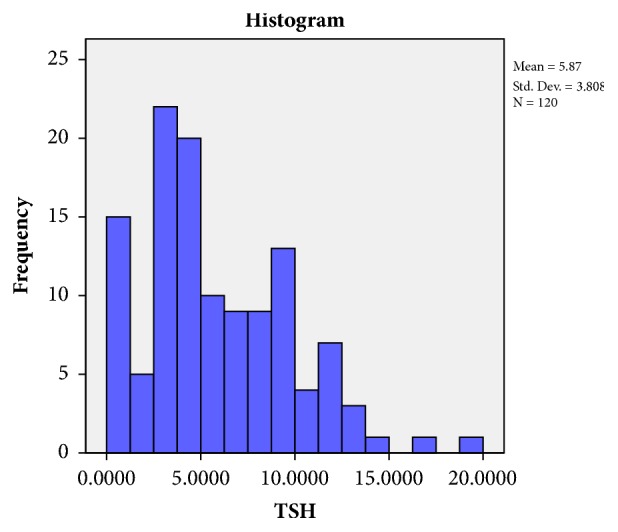
Distribution of TSH in blood in study population (n=168).

**Figure 2:**
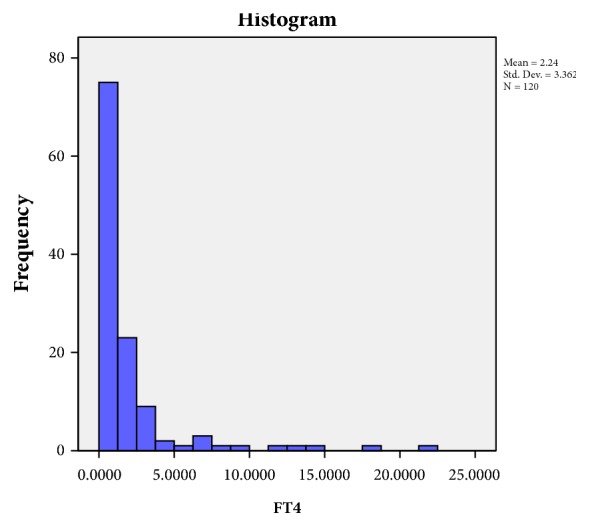
Distribution of FT4 level in study population (n=168).

The study revealed that 168 patients (33.39% of thyroid dysfunction and 15.27% of total population) had the biochemical features suggestive of subclinical hypothyroidism (defined by no symptoms or clinical features of hypothyroidism but biochemically TSH level in the range of above 5 miu/ml but below 10 miu/ml with normal FT4 level). Normal range for FT4 level in blood is 0.7-1.24 *μ*g/dl (standardized for all value). Figures 1 and 2 showed the distribution of FT4 and TSH level in those 168 patients. To know the prevalence of autoimmunity in thyroid dysfunction anti-TPO test was done. The study revealed 14 patients out of 168 patients with subclinical hypothyroidism positive for antithyroid peroxidase (TPO) antibody positivity (0.02% of total thyroid abnormality). Further subanalysis of those 168 patients with thyroid dysfunction has been depicted in Table 3. Average TSH level was found to be 7.67, 6.13, and 9.38 in microunits/ml in STEMI, NSTEMI, and Unstable Angina group, respectively. Average FT4 level was found to be 0.67, 0.96, and 0.88 *μ*g/dl in STEMI, NSTEMI, and UA group, respectively. There was no statistical significance p value in terms of TSH level in blood. 100% of the patients were smoker in all the three groups. Anti-TPO positivity has been shown in Table 3 for each group of patients. Single vessel diseases were more common in all the 3 groups in those 168 patients. The impact of thyroid dysfunction between three groups is found to be not significant (p value = 0.088) in this study.

## 7. Discussion

The prevalence of hypothyroidism is estimated to be 2% to 4% and increases with advancing age [7]. In contrast to the dramatic clinical signs and symptoms of hyperthyroidism, the cardiovascular findings of hypothyroidism are more subtle [8]. This study revealed FT4 level in blood has little value in screening of thyroid dysfunction as most of the patients were found to have normal level of free l-thyroxine in all the three groups. But TSH level is significantly abnormal in large number of patients reflecting underlying thyroid abnormality in majority of sample population.

Prevalence of subclinical hypothyroidism is found to be quite high (15.27%) in ACS patients. This high prevalence was in contrast to several other studies done outside India [9] which reflects 2.3-6.7% prevalence. Considering the high prevalence of thyroid dysfunction in Indian subcontinent this high association might seem appropriate. The male prevalence is much more in this study although subclinical hypothyroidism is said to be more common in females [10]. Smoking definitely has a strong association in the causation of cardiovascular diseases as evident from this study. Mild degrees of bradycardia, diastolic hypertension, a narrow pulse pressure and relatively quiet precordium, and decreased intensity of the apical impulse are all characteristics [11]. Interestingly almost all patients were tachycardic with heart rate more than 100/minutes irrespective of baseline thyroid dysfunction. Diastolic hypertension was also not noted in this study. There was no significance difference in terms of LVEF, angiographic pattern, and outcomes in the three groups comparing those with and without subclinical hypothyroidism. Hypothyroidism also increases total and low-density lipoprotein (LDL) cholesterol in proportion to the rise in serum TSH levels [12]. Lipid profile was found to be deranged in this study in all the three groups as evident in Table 1. There was no significant difference in mortality in patients with thyroid dysfunction in the three groups. Despite lack of satisfactory data in subclinical hypothyroidism patients having ACS treatment with levothyroxine may be found fruitful as it might lower cholesterol lever and can improve cardiac contractility.

## 8. Conclusion

This study is the first of its kind in Eastern India, showing high prevalence of subclinical hypothyroidism in ACS patients. Therefore treatment algorithm should be initiated in those group of patients. There are no current consensus guidelines regarding the treatment in this settings so far. The causal association between abnormal thyroid hormone levels and ACS is not fully understood. Different studies support a biologically plausible role for hypothyroidism increasing the risk of atherosclerotic cardiovascular diseases. Though this study does not demonstrate any significance difference in terms of mortality and other parameters (LVEF, angiographic findings) long term follow-up may differ.

## Figures and Tables

**Table 1:** Showing results of baseline demographics in all 3 groups.

**Baseline Parameter**	**STEMI**	**NSTEMI**	**Unstable Angina**
**Patients (n=1100)**	493(44.81%)	403(36.63%)	204(18.54%)
**Age (average) (yrs)**	62.34	58.98	53.76
**Sex (Male/Female)**	301/102	298/105	101/103
**Blood Pressure (average)**	140/92	130/90	150/85
**Heart Rate (average/min)**	106	104	101
**Cheat pain present**	100%	100%	100%
**Hemodynamic instability**	63	41	23
**LVEF % (average)**	46	49	51
**CAG-single vessel disease (significant)**	110	137	93
**CAG-Multivessel disease**	360	254	101
**LDL (mg/dl)**	125	119	129
**HDL (mg/dl)**	41	42	46
**TG (mg/dl)**	145	187	176
**Smoking (number of patients)**	476	365	201

**Table 2:** Showing distribution of angiographic pattern in all the groups.

	STEMI	NSTEMI	UA
LAD	44.64286	47.91666	47.500
LCX	26.78571	20.83333	22.500
RCA	28.57143	31.250	30.000

**Table 3:** Showing characteristics in subclinical hypothyroidism patients (n=168).

	STEMI	NSTEMI	Unstable Angina
Patients population (n=168)	60 (35.71%)	62 (36.90%)	46 (27.38%)
Serum TSH (*µ*IU/ml)	7.67	6.13	9.38
Serum FT4	0.67	0.96	0.88
Single Vessel Disease (LAD, RCA, LCX)	43 ( 30, 10, 3)	40 ( 32, 6, 2)	33 ( 16,15, 2)
Anti TPO Positive	4	2	8

## Data Availability

The data used to support the findings of this study are available from the corresponding author upon request.
